# Review of core stability exercise versus conventional exercise in the management of chronic low back pain

**DOI:** 10.4314/ahs.v22i4.19

**Published:** 2022-12

**Authors:** Obinna Dickson Nwodo, Peter Olanrewaju Ibikunle, Nnenna Linda Ogbonna, Kenneth Umezulike Ani, Arinze Christain Okonkwo, Chinenye Joy Eze, Chukwudi Uchenna Onwudiwe, Godwin Uroko Ezeja, Ifeoma Adaobi Maduanusi

**Affiliations:** 1 Department of Physiotherapy, Alex Ekwueme Federal University Teaching Hospital, Abakaliki, Ebonyi State, Nigeria; 2 Department of Medical Rehabilitation, Faculty of Health Sciences and Technology, College of Health Sciences, Nnamdi Azikiwe University, Nnewi Campus, Anambra State, Nigeria; 3 Department of Medical Laboratory, Enugu State University of science and Technology Teaching Hospital, Enugu, Enugu State, Nigeria; 4 Department of Pharmaceutical Science, University of Nigeria, Nsukka, Enugu State, Nigeria

**Keywords:** Core stability exercise, conventional exercise, chronic low back pain

## Abstract

Exercise has been proven to be effective in the management of chronic low back pain. Over the years, core stability exercise (CSE) has gained popularity however there is lack of consensus on the best exercise treatment.

**Aims:**

To review the effectiveness of core stability exercises or conventional exercises in the management of chronic low back pain (CLBP).

**Methods:**

This study is a systematic review of randomized clinical trials which examined studies regarding core stability and conventional exercise by using Google scholar, Medline, PEDro and Cochrane from 2010 to 2021. The Methodological quality was evaluated using the PEDro scale. The included studies randomized participants into two different exercise groups.

**Results:**

From the 58 potentially relevant trials, a total of 14 trials were included in the current analysis. The data indicated that core stability exercise was better than conventional exercise for short term pain relief. Ten studies included self reported back specific functional status, and compared to conventional exercise, core stability exercise resulted in significant improvement in function.

**Conclusion:**

Compared to conventional exercise, core stability exercise is more effective in pain reduction and improved physical function in individuals with CLBP in the short term however, only two trials carried out follow-up assessments post intervention.

## Introduction

Low back pain (LBP) is the single biggest cause of years lived with disability worldwide and a major problem to global health system.^1^ Low back pain by definition is pain in the region between the lower margin of the 12^th^ rib and the gluteal folds with or without distal radiation to the lower extremity.^2^ In western countries, the estimated lifetime prevalence of LBP ranges from 30% to 79.2% whilst the lifetime prevalence of low back pain in Africa ranged from 28% to 74% and was almost correspondent to the rates in the Western societies.^3^ There is evidence that the prevalence and costs of LBP are rising around the globe due to ageing and expanding world population.^4^ More so, it has been asserted that the risk of low back pain is twice as high once a history of the condition have been established.^5^ Despite this, the most appropriate intervention to LBP remains elusive.^6^ The causes of LBP are complex and not clearly understood; although some risk factors are implicated.^7^ Individuals with LBP commonly present with decreased flexibility in the lumbar region and functional status. The latest clinical practice guidelines recommend that patients remain physically active, as long period of physical inactivity contributes negatively to recovery and the general wellbeing.^8^ Many exercise techniques have been developed for the treatment of CLBP. Their aims are pain decrease, muscular strengthening in flexion or extension.^9^ Core stability training is fast becoming a popular rehabilitation regimen in the management of CLBP, as it involves the restoration of the ability of the neuromuscular system to control and protect the spine from injury or re-injury.^10^,^11^ Bronfort et al^12^ found that supervised core stability training improves pain severity, disability level and general health after some weeks of treatment. More so, a meta-analysis by Wang et al 13 found that core stability exercises produced better outcomes than routine exercise therapy during the initial three months of intervention for low back pain. Conventional exercise has been in use for long period of time and also has been established to be effective in pain reduction and improved strength.^14^ Consensus on the most effective types of exercises for the treatment of CLBP has not been reached yet. Hence, the objective of the review was to investigate the effectiveness of core stability training and conventional exercise in patients with low back pain measured in randomized controlled trials on pain and disability outcomes.

## Methodology

### Study Design

We conducted and reported this systematic review according to the PRISMA guidelines, with a protocol defined a priori. We identified randomized controlled trials (RCTs) by electronically searching the following databases: Physiotherapy Evidence Database (PEDRo), Medline, Cochrane, Google scholar, and others. Furthermore, we conducted a hand-searching of the reference lists of the articles found from the databases and journals to identify additional relevant articles. Briefly, the following medical subject headings (MeSH) were included: low back pain, sciatica, lumbo-sacral region, exercise, and chronic pain. We restricted our searches to only studies published in English language between the years 2010 to 2021. We arrived at the decision so as to eliminate the cost of language translations and to ensure that the identified and included articles in this review would not be studies that are too staled. The keywords used were RCTs, double-blind method, single-blind method, random allocation, pelvic girdle pain, motor control, exercise therapy, stability, stabilization, traditional exercise, conventional exercise, specific exercise, and physical therapy. We removed duplicates that were identified in multiple database searches. Reference lists of the included articles were also manually searched for relevant studies. All the literature searches in the afro-mentioned databases and journals were performed between March and August, 2021.

### Inclusion Criteria

Literature search was conducted through several steps. First, the objective of this study was defined with population, intervention, comparison, and outcome (PICO) techniques. These techniques were determined to establish the eligibility criteria for this study as follows.

### Types of studies

Only RCTs examining the effects of core stability exercise versus conventional exercise for the treatment of patients with chronic LBP were included. Types of participants: We included articles with both female and male subjects (over 18 years of age) who had chronic LBP (longer than 3 months). We excluded articles that included participants with LBP evoked by specific conditions or pathologies.

### Types of interventions

We included articles that compared a control group, which received conventional exercise, and treatment group, which received core stability exercise training. A core stability training program could be described as the reinforcement of the ability to ensure stability of the neutral spine position.^10^

### Types of outcome measures

The primary outcomes of interest were pain intensity and back-specific functional status.

### Selection of Studies

Two reviewers (Nwodo Obinna and Onwudiwe Chukwudi) were used for the pre-specified criteria to screen for relevant titles, abstracts and full papers. Articles were removed once inclusion criteria were not met. In conflicting situations, a third reviewer (Ogbonna Linda Nnenna) was consulted

### Exclusion Criteria

Exclusion criteria were listed as follows: title keywords unrelated to research topics; unclear articles; and incomplete study, study protocols, abstract, and review articles.

### Data Extraction

We extracted the following data from the included articles: study design, subject information, description of interventions between the core stability exercise and conventional exercise group, follow-up period, and outcome measures (Table 1). These data were then compiled into a standard table. The two reviewers (Nwodo Obinna and Onwudiwe Chukwudi) who selected the appropriate studies also extracted the data and evaluated the risk of bias. It was necessary to consult an arbiter (Ogbonna Linda Nnenna) to reconcile any disagreements.

**Table 1:** Characteristics of included studies

	Authors and country	Variables	Study population	Sample size	Sourced databases	Participants characteristics	Aims of the study	Main finding
1.	Javadian et al^17^ (2012) Iran	Pain and function	Lumbar Segmental Instability, a subgroup of non specific low back pain	N = 30 Core stability group: 15. Convention al group: 15.	Cochrane Library	**Core stability** **exercise group:** Bracing and hollowing treatment in supine, Bridging, kneeling, sitting and standing positions. **Routine exercise** **group:** Single and double leg knee to chest, Bridging, Interval lower limb raising, supine cycling, heel and leg slide, lower abdominal crunch	The purpose of the study was to determine the effect of core stabilization exercises on pain, functional disability and muscle endurance in patients with Lumbar segmental instability.	Core stabilization exercises plus routine exercises are more effective than the routine exercise alone in decreasing pain, increasing functional disability and muscle endurance of patients with signs and symptoms of lumbar segmental instability
2.	You et al^18^ (2013) Republic of South Korea	Pain and Physical function	Chronic low back pain	N = 40 Core stability group: 20	MEDLINE Cochrane	**Core stability** **group**: adding ankle dorsiflexion to drawing in the abdominal wall.	To identify the effect of a novel augmented core	After intervention, core stability exercise group showed significant
2.	You et al18 (2013) Republic of South Korea	Pain and Physical function	Chronic low back pain	N = 40 Core stability group: 20 Convention al group: 20	MEDLINE Cochrane	**Core stability** **group**: adding ankle dorsiflexion to drawing in the abdominal wall. **Conventional** **exercise** **group:** gentle massage, Passive range of motion, treadmill gait training.	To identify the effect of a novel augmented core stability exercise technique on physical function, pain and core stability in patients with chronic low back pain.	After intervention, core stability exercise group showed significant greater improvement at two months compared with convent ional exercise group
3.	Ebrahimi et al^19^ (2014) Iran	Pain, function, abdominal and back muscle endurance	Chronic low back pain due to disc herniation	N = 30 Core stability group: 15. Convention al treatment group: 15.	Medline Cochrane	**Core stability** **group:** received d ifferent stretching and strengthening exercises. **Conventional** **treatment** **group:** received traditional Physiotherapy treatment.	The aim of the study was to investigate the effect of core stabilization exercises on low back pain and abdominal and back muscle endurance in patients	Core stabilization exercises in improving lo w back pain, abdominal and back muscle endurance in patients with chronic low back pain caused by disc herniatio
3.	Ebrahimi et al^19^ (2014) Iran	Pain, function, abdominal and back muscle endurance	Chronic low back pain due to disc herniation	N = 30 Core stability group: 15. Convention al treatment group: 15.	Medline Cochrane	**Core stability** **group:** received different stretching and strengthening exercises. **Conventional** **treatment** **group**: received traditional Physiotherapy treatment.	The aim of the study was to investigate the effect of core stabilization exercises on low back pain and abdominal and back muscle endurance in patients with chronic low back pain caused by disc Herniation.	Core stabilization exercises in improving lo w back pain, abdominal and back muscle endurance in patients with chronic low back pain caused by disc herniatio n have been effective.
4.	Cho et al 20 (2014) Republic of Korea	Pain, range of motion and function	Chronic low back pain	N = 30 Core stability group: 15 Convention al group: 15	Google scholar Cochrane	**Core stability** **exercise group:** Were administered in three parts; warm up, conditioning and cool down as described in brills book(**ref**) **Conventional** **exercise group:** r	The study aimed to identify the effects of the CORE exercise program on pain and active range of motion (AROM) in patients	The study demonstrated that the core exercise program is effective in decreasing pain and increasing AROM in patients with
4.	Cho et al 20 (2014) Republic of Korea	Pain, range of motion and function	Chronic low back pain	N = 30 Core stability group: 15 Convention al group: 15	Google scholar Cochrane	**Core stability** **exercise group:** Were administered in three parts; warm up, conditioning and cool down as described in brills book(**ref**) **Conventional** **exercise group:** received routine care	The study aimed to identify the effects of the CORE exercise program on pain and active range of motion (AROM) in patients with chronic low back pain..	The study demonstrated that the core exercise program is effective in decreasing pain and increasing AROM in patients with chronic low back pain.
5.	Alp et al 21 (2014). Turkey	Pain, Quality of life, abdominal and back muscle endurance	Chronic low back pain	N = 48 females Core stability group: 24. Home based conventional exercise group: 24.	Cochrane Library Google scholar	**Core stability** **exercise group:** Were administered in three parts; warm up, stretching and stabilization exercises **Conventional** **exercise group:** received routine care	The purpose of the study was to investigate the efficacy of core-stabilization exercise (SE) and to compare it with home-based conventiona l exercise (HE) in patients with chronic low-back pain (LBP)	Though both of the exercise programs were both found to be effective concerning the areas of pain, endurance, function, and daily living in patients with chronic LBP, the SE group was superior to the HE group in the endurance of dorsal extensors and in the improvement of physical role limitation exercises
6.	Cho et al 22 (2015) Republic of Korea	Pain, functional disability and lumbar lor dosis angle.	Chronic low back pain	N = 30 Core stability group: 15. Convention al group: 15.	Cochrane PEDRO	**Core stability** **group**: received lumbar stabilization exercises **Conventional** **group:** Received hot pack, Interferential current therapy and ultrasound therapy.	The study examined the effects of lumbar stabilization exercises on the functional disability and lumbar lord osis angles in patients with chronic low back pain.	Lumbar stabilization exercise is more effective than conservative treatment for improving functional disability and lumbar lordos is angles.
7.	Reddy et al^23^ (2015) India	Pain and function	Chronic mechanical low back pain	N = 40 Core stability group: 20. Conventional group: 20.	Medline Cochrane PEDRO	**Conventional** **group:** Supine lying – Leg lifts Abdomin al crunches in crook lying. Prone lying – Leg lifts Prone lying – Trunk lifts. **Core stability** **group:** Stabilization exercises using medicine ball	To compare the outcome of conventional and core stabilization exercises in chronic mechanical low back pain.	Core stabilization group registered a significant improvement when compared to conventional back care exercises in improving function and relieving pain when co mpared to conventional back care exercises in improving function and in relieving pain.
8.	Akhtar et al^24^(2017) Pakistan	Pain and physical function	Non specific low back pain	N = 120 Core stability group: 60. Convention al group: 60.	Google scholar, PEDro, Cochrane	**Core stability** **group:** received only core stability eg. Multi fidus exercise, Frontal & Side Plank exercise, Pelvic floor exercises, Wobble board oblique twist, etc **Conventional** **group:** Received routine physical therapy eg back, h amstring, calf and hip flexors stretches, Abdominal curl up exercises etc along with ultrasound and Transcutaneo us electrical nerve stimulation.	The study was designed to compare the effectivenes s of specific stabilization exercises with routine physical therapy exercise provided in patients with nonspecific chronic mechanical low back pain.	Core stabilization exercise is more effective than routine physical therapy exercise in terms of greater reduction in pain in patients with non-specific low back pain
9.	Ali et al 25 (2017). Pakistan	Pain and functional disability	Chronic low back pain	N = 40 Core stability plus conventional Physiotherapy group: 20. Conventional Physiotherapy group: 20.	Cochrane Library	**Core stability** **exercise plus** **conventional** **Physiotherapy** **group:** TENS, Hot Pack, spinal mobilization, Stretching exercises and Lumbar Stabilization Exercises **Conventional** **Physiotherapy** **group:** TENS, Hot Pack, spinal mobilization and Stretching exercises.	To determine the effect of lumbar stabilization exercises on pain, range of motion and functional disability in the management of chronic low back pain.	Lumbar stabilization exercises in addition to conventional physiotherapy were found more effective in chronic low back pain management as compared to conventional physiotherapy alone in terms of reducing pain and functional dis ability.
10.	Ghorbanpour et al^26^ (2018). Iran	Pain, range of motion and functional disability	Chronic non specific low back pain	N = 34 Mcgill core stability group: 17. Conventional Physiotherapy group: 17.	Cochrane Library Google Scholar	**Core stability** **group:** Curl up, Side Bridge, Bird Dog with one hand or one foot and one hand and the opposite leg. **Conventional** **Physiotherapy** **group:** single and double knee to chest, prone lying with pillow with one leg sliding, cycling in supine and bridging exercises.	To compare the effects of “McGill stabilization exercises” and “conventional physiotherapy” on pain, functional disability and active back flexion and extension range of motion in patients with chronic nonspecific low back pain.	The results indicated that McGill stabilization exercises and conventional physiotherapy provided approximatel y similar improvement in pain, functional disability, and active back range of motion in patients with chronic non-specific low back pain. However, it appears that McGill stabilization exercises provide an additional benefit to patients with chronic non-specific low back, especially in pain and functional disability improvement
11.	Noormoham madpour et al^27^ (2018). Iran	Pain, quality of life and function disability	Chronic low back pain	N = 38 female nurses Core stability group: 18 Conventional group: 18	Google scholar Cochrane	**Core stability** **group**: Received mult i-step core stability exercise program **Conventional** **exercise** **group:** Not reported	To evaluate the effects of a multistep core stability exercise program in nurses with chronic low back pain (CLBP).	This study showed that a multi-step core stability exercise program is a helpful treatment option for improving quality of life and reducing disability and pain in female nurses with CLBP.
12.	Waseem et al^28^ (2018). Pakistan	Pain, function, abdominal and back muscle endurance	Chronic low back pain due to disc her niation	N = 30 Core stability group: 15. Conventional treatment group: 15.	Medline Cochrane	**Core stability** **group:** receive d different stret ching and strengthening exercises. **Conventional** **treatment** **group:** receive d traditional Physiotherapy treatment.	The aim of the study was to investigate the effect of core stabilization exercises on low back pain and abdominal and back muscle endurance in patients with chronic low back pain caused by disc Herniation.	Core stabilization exercises in improving low back pain, abdominal and back muscle endurance in patients with chronic low back pain caused by disc herniation have been effective.
13.	Akter et al^29^ (2020). Bangladesh	Pain, and functional disability	Chronic low back pain	N = 30 Segmental stabilization exercise with conventional therapy group: 15 Conventional therapy group: 15	Google scholar Cochrane	**Segmental** **stabilization** **exercise with** **conventional** **therapy** **group:** Received stabilization exercises alongside conventional therapy. **Conventional** **therapy** **group:** received conventional therapy	To identify whether segmental stabilization exercise with conventional therapy program or only conventional therapy program is more effective for the treatment of chronic low back pain patients.	This research showed that segmental stabilization exercises combined with conventional therapy was more effective than only conventional therapy for patients with chronic low back pain.
14.	Yangma et al^30^ (2021) India	Pain, Range of motion and functional disability	Mechan ical lo w back pain	N = 40 Core stability group: 20 Conventional exercise group: 20	Google scholar	**Core stability** **exercise** **group:** administ ered stabilization exercises using swiss ball **Conventional** **exercise group:** received Crook lying with Crunches, supine with leg lifts, Prone lying with leg lifts, Prone with trunk lifts	The purpose of this study is to compare the effectiveness of core muscles activation over conventional exercises in reduction of pain and increasing Range of Motion of trunk in subjects with Mechanical low back pain	It is concluded that subjects in the group who received Core muscles activation exercises are more effective as compared to the group who received Conventional exercise in reducing pain, increasing the ROM and improving the disability.

### Critical Appraisal Instruments

The Physiotherapy Evidence Database (PEDro) scale was the critical appraisal instrument used in this study. The tool comprised eleven elements, and each element required a dichotomous yes/no response: 1 point was given to yes and 0 was allocated to no. The total score for the PEDro scale was 10. PEDro scores were excellent (9–10), good (6–8), fair (4–5), and poor (less than 4) Foley et al.^15^

### Risk of bias assessment

The risks of bias within and across the study were assessed by using the Cochrane Effective Practice and Organization of Care risk of bias tool (Cochrane Effective Practice and Organization of Care Group).^16^ This tool comprised nine items, namely, random sequence generation, allocation concealment, similar baseline outcome, similar baseline characteristics, incomplete outcome data, blinding, contamination, selective outcome reporting, and other biases. All of the items were given a score of high risk, low risk, or unclear (Table 2).

**Table 2:** Appraisal of risk of bias, according to Cochrane Effective Practice and Organization of Care risk of bias tool

Criterion/articles	Random sequence generation	Allocation concealment	Similar baseline outcome	Similar baseline characteristics	Incomplete outcome data	Blinding	Contamination	Selective outcome reporting	Other bias
Javadian et al^17^ 2012	Low	Unclear	Low	Low	Low	Unclear	High	Unclear	Unclear
You et al^18^ 2013.	Low	Low	Low	Low	Low	Low	High	Unclear	Unclear
Ebrahimi et al^19^ 2014	Unclear	Unclear	Unclear	Low	Unclear	Unclear	High	Unclear	Unclear
Cho et al^20^ 2014.	Low	Unclear	Low	Low	Low	Unclear	Low	Unclear	Unclear
Alp et al^21^ 2014.	Low	Low	Low	Low	Low	Low	Low	Unclear	Unclear
Cho et al^22^ 2015	Unclear	Unclear	Low	Low	Low	Unclear	Low	Low	Unclear
Reddy et al^23^ 2015	Low	Unclear	Unclear	Unclear	Low	Unclear	High	Low	Unclear
Akhtar et al^24^ 2017.	Low	Low	Low	Low	Low	Low	Low	Low	Unclear
Ali et al^25^ 2017.	Low	Unclear	Low	Low	Low	Unclear	Low	Low	Unclear
Ghorbanpour et al^26^ 2018	Low	Low	Low	Low	Low	Low	Low	Low	Unclear
Noormohammadpour Et Al^27^ 2018.	Low	Low	Low	Low	Low	Low	Low	Low	Unclear
Waseem et al^28^ 2018.	Low	Low	Low	Low	Low	Low	Low	Low	Unclear
Akter et al^29^ 2020	Low	Low	Low	Low	Low	Low	High	Low	Unclear
Yangma et al^30^ 2021.	Low	Low	Low	Low	Low	Low	Low	Unclear	Unclear

## Results

### Search Results

**Figure 1:**
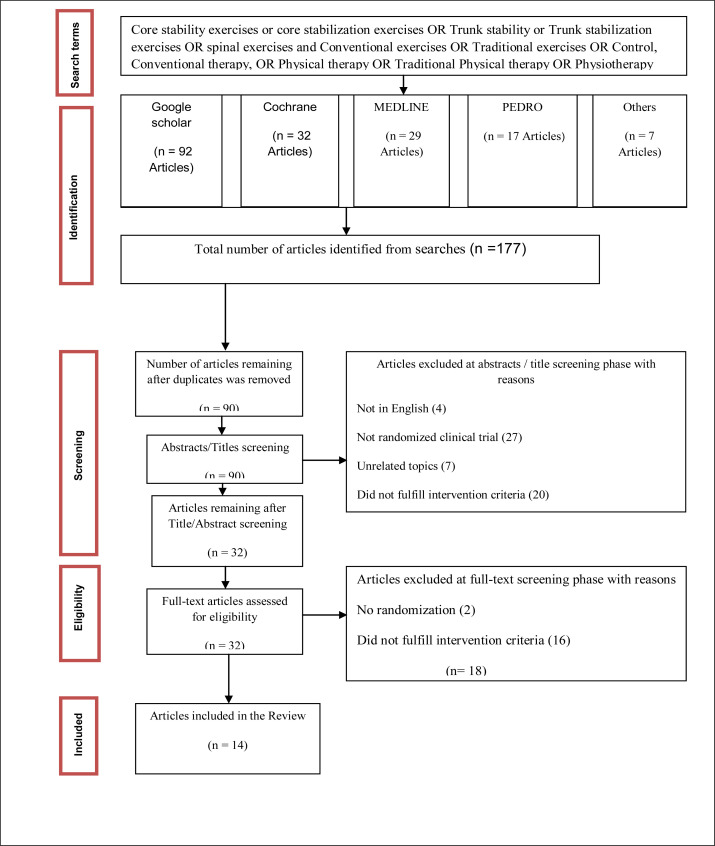
PRISMA diagram of the search strategy and study selection processes.

The process of identifying eligible studies was outlined in figure 1. One hundred and seventy-seven records were initially identified through the Cochrane, Medline, Google scholar, PEDro and others. Of these, 58 potentially eligible articles were included based on their title and abstract. After reviewing these 58 potential articles, only 14 articles fulfilled the inclusion criteria.^17^–^30^ The remaining 44 articles were removed because the trials included was not randomized, did not compare core stability exercise with conventional exercise, or the original data were not available from the authors.

### Included studies

Studies were included if they were RCTs on efficacy of core stability exercise and conventional exercises in CLBP management, published in English between 2010 and 2021. Included patients were between the age of 18 and 60 years. Below are the individual characteristics of the included studies.

### Intervention

CSE is the exercise that involves the spine and core muscles (mostly the transversus abdominis or multifidus), where the core muscles are tightened to while the spine is being stabilized and then progressed to functional activity. 24 Conventional exercises are traditional exercises that are not specifically targeted to the core muscle of the spine.^24^

### Data synthesis/extraction

Data synthesis involved the combination and summary of findings of the studies selected for the review. The synthesis of the data was done by the descriptive synthesis using the extraction form designed by the reviewer to outline characteristics of the studies.

### Data analysis

The selected studies for the review were appraised using the PEDro scale. The methodological quality of all selected studies for the review was strictly assessed by two independent reviewers (Nwodo Obinna and Onwudiwe Chukwudi) with blinding.

### Outcome measures

#### Pain Intensity

Twelve out of the fourteen included study assessed pain intensity using visual analogue scale, of the other two trials, one examined pain intensity using Numerical Pain Rating Scale and the other did not assess pain intensity (Table 4).

**Table 4:** Outcome measure and time point

References	Outcome measure	Statistical tests	Time point
Javadian et al^17^, 2012	Visual Analogue scale Modified schober's test Oswestry disability index Endurance time for trunk flexors and extensors	Intra correlation coefficient, Standard error of measurement, Kolmogorov Smirnov test, Repeated measure ANOVA, Independent t-test.	8 weeks (Intervention) 3 months (Follow up)
You et al^18^, 2012	Visual Analogue scale Pain disability index Pain rating scale Active straight leg raise Oswestry disability index Rolland Morris disability questionnaire	Repeated measure ANOVA Independent t-test Post hoc Bonferroni test	8 weeks (Intervention) 2 months (Follow up)
Ebrahimi et al^19^, 2014	Visual analog scale Back extensor muscles endurance Abdominal muscle endurance	Kolmogorov Smirnov test, ANCOVA, Dependent t-test	8 weeks
Cho et al 20 2014	Visual analogue scale Pain pressure threshold using an algometer	Independent t test Descriptive Statistics	4weeks
Alps et al^21^ 2014	Visual analogue scale Roland Morris Back Pain Disability Questionnaire Short Form 36 health survey Kraus-weber test Sorensen test	Descriptive Statistics Shapiro-wilk test Mann Whitney U test Wilcoxon signed rank test Student test	6 weeks
Cho et al^22^ 2015	Visual analogue scale Modified Oswestry Disability Index (ODI)	Paired t test Independent t test	6 weeks
Reddy et al^23^ 2015	Visual Analogue scale Roland Morris Back Pain Disability Questionnaire Lumbar range of motion	One way ANOVA, Independent t-test.	6 weeks
Akhtar et al^24^ 2017	Visual Analogue scale	Friedman ANOVA Mann Whitney U test	8 weeks
Akhtar et al^24^ 2017	Visual Analogue scale	Friedman ANOVA Mann Whitney U test Wilcoxon test	8 weeks
Ali et al^25^ 2017	Numerical pain rating scale Oswestry disability index Lumbar range of motion Trunk flexors and extensors time	Descriptive statistics Mann Whitney U test Wilcoxon test	2 weeks
Ghorbanpour et al^26^ 2018	Visual analogue scale Quebec low back pain disability scale Lumbar range of motion using inclinometer	Independent t test Descriptive Statistics Paired t test	2 weeks
Noormohammadpour et al^27^ 2018	Visual analogue scale Roland Morris Back Pain Disability Questionnaire Short Form 36 health survey Lateral abdominal muscle diameter via Ultrasound	ANCOVA Paired t test Descriptive Statistics	8 weeks
Waseem et al^28^ 2018	Modified Oswestry Disability Index (ODI)	Friedman ANOVA, Mann Whitney U test	6 weeks
Akter et al 29 2020	Visual Analogue scale Descriptive Statistics 6 weeks
Akter et al 29 2020	Visual Analogue scale Oswestry disability Index Oxford muscle strength scale	Descriptive Statistics Mann Whitney U test Wilcoxon test	6 weeks
Yangma et al^30^ 2021	Visual Analogue scale Oswestry disability Index Lumbar range of motion using gonimetry	Descriptive statistics Paired sample t test Independent sample test	12 weeks

#### Disability

Ten studies included assessed disability level. Of these, seven used Oswestry Disability Index; two used Rolland Morris Disability Questionnaire and one utilized Quebec Low Back Pain Disability Index (Table 4).

### Duration of intervention

The duration of the interventions in the studies included in this review were about 30 min per each session, twice a week for at least two weeks to twelve weeks.

### Dropouts

Of the fourteen studies included, five reported dropout of participants during the study period (Table 5).

**Table 5:** Duration of intervention and withdrawal

First author	Duration of intervention (week)	Follow up intervention	Number of participant withdrawal	Reasons for the withdrawal
Javadian et al 17 2012	8 weeks (twice per week)	3 months	None	None
You et al^18^ 2013	8 weeks (thrice per week)	Two months	None	None
Ebrahimi et al^19^ 2014	8 weeks (thrice per week) No follow up	None	None
Cho et al^20^ 2014	4 weeks (thrice per week)	No follow up	None	None
Alp et al^21^ 2014	6 weeks (thrice per week)	3 months	None	None
Reddy et al^22^ 2015	6 weeks (thrice per week)	None	None	None
Cho et al^23^ 2015	6 weeks (thrice per week)	None	None	None
Akhtar et al 24 2017	6 weeks (once per week)	No follow up	Twelve (12)	1.Lost to death: 2 2.Moved to another city: 6 3.Discontinued intervention: 1 4.Unable to make time commitment : 3
Ali et al^25^ 2017	2 weeks (four times per week)	No follow up	Two (2)	None
Ghorbanpour et al^26^ 2018	6 weeks (thrice per week)	No follow up	Four (4)	Unwilling to continue due to family related problem
Noormohammadpour et al^27^ 2018	8 weeks	No follow up	Sixteen (16)	1. Two became Pregnant. 2. Two moved out of the city. 3. Four had problem with work schedule interfering with participation. 4. Two had retired. 5. Six had withdrawn themselves to find another treatment option.
Waseem et al^28^ 2018	6 weeks (twice per week)	No follow up	Twelve (12)	1.Lost to Death: 2 2. Moved out of the city: 6 3. Unable to make time commitment: 3 4. Unable to continue with intervention: 1
Akter et al^29^ 2020	4 weeks (three times per week)	No follow up	None	None
Yangma et al^30^ 2021	12 weeks	No follow up	None	None

### Collating, summarizing and reporting the results. Descriptive summary of the results

Fourteen peer reviewed studies were included in this review. The studies were published in seven countries; Pakistan, Iran, Republic of Korea, Turkey, India, Republic of South Korea and Bangladesh.

### Research designs used by the included studies

The research designs adopted by the included studies were randomized clinical trial study (Table 6).

**Table 6:** Characteristics of the included studies

Author	Study design	Sample size	Mean age	Experimental and control group	Intervention time (week)	Pedro score
Javadian et al^17^ 2012	RCT	CSE grp: 15 Routine grp:15	Nil Nil	Stability exercise plus routine exercises group versus routine exercises group	Two times per week for eight weeks	5
You et al^18^ 2013	RCT	CSE grp: 20 Con.PT grp 20	50.35 51.30	Core stability techniq ue group versus Conventional Physical therapy group	40 minutes, three times a week for eight weeks	10
Ebrahimi et al^19^ 2014	RCT	CSE grp: 15 Con.grp: 15	48.55	Core stability exercise group versus Convent ional therapy group	30 minutes, three times a week for eight weeks	5
Cho et al^20^ 2014	RCT	CSE grp: 15 Control grp: 15	38.1 36.5	Core exercise program versus Contr ol group	30minutes, three times per week for four weeks	6
Alp et al^21^ 2014	RCT	CSE grp: 24 Home based Con. Exer: 24	48 51	Core stabilization exercise group versus Home based conventional exercise group	60 minutes, Three times per week for six weeks	9
Reddy et al^22^ 2015	RCT	CSE grp: 20 Con. Back Exer grp: 20	Nil Nil	Core stabilization exercise group versus Conventional back exercise group	30minutes, three times a week for six weeks	5
Cho et al^23^ 2015	RCT	CSE grp: 15 Con. Back Exer grp: 15	Nil Nil	Core stabilization exercise group versus Conventional back exercise group	30minutes, three times a week for six weeks	6
Akhtar et al^24^ 2017	RCT	CSE grp:60 Routine Exer. grp:30	46.39 45.5	Core stabilization exercise group versus the routine exercise group	Once per week for six weeks	9
Ali et al^25^ 2017	RCT	LSE grp: 20 Con.PT grp : 20	31.75 46	Lumbar stabilization exercise group versus Conventional Physiotherapy group	Four times per week for two weeks	7
Ghorbanpour et al^26^ 2018	RCT	Mcgill Stability Exer grp: 15 Con.PT grp: 15	23.8 20.90	Mcgill stabilization exercise group versus Conventional Physiotherapy group	Three times a week for six weeks	8
Noormohammadpour et al^27^ 2018	RCT	Multi-step Core stability grp: 10 Control grp: 10	43.3 41.3	Multi-step core stability exercise group versus Control group	Eight weeks	9
Waseem et al^28^ 2018	RCT	CSE grp: 53 Routine PT grp: 55	46.39 45.5	Core Stabilization exercise program versus Routi ne Physical therapy group	Two times per week for six weeks	8
Akter et al^29^ 2020	RCT	SSE plus Con. therapy : 15 Con. therapy: 15	36.40 39.27	Segmental stabilization exercise plus Con. Therapy group versus Conventional therapy group	30 minutes, Three times per week for four weeks	10
Yangma et al^30^ 2021	RCT	CSE grp: 20 Con. Exer grp: 20	Nil Nil	Core stability exercise grp versus Conventional exercise grp.	Twelve weeks	6

**KEY**

CSE grp: Core stability exercise group

LSE: Lumbar stabilization exercise

SSE: Segmental stabilization exercise

RCT: Randomized clinical trial

Con: Conventional

PT: Physiotherapy

### Core Stability Exercise versus Conventional Exercise on Pain Intensity

In total, fourteen trials were included in the study, twelve of which assessed pain intensity using visual analogue scale^17^–^24^, ^26^, ^27^, ^29^, ^30^ one of the two trials examined pain intensity using Numerical Pain Rating Scale^25^ and the other trial did not evaluate pain intensity^28^. The data indicated that core stability exercise was better than conventional exercise for short term pain relief. Only two trials out of the fourteen evaluated pain intensity at three months post intervention, and found core stability exercise to be more beneficial in pain reduction than conventional exercise.

### Core Stability Exercise versus Conventional Exercise on disability

Eleven studies included self reported back specific functional status.^17^, ^18^, ^21^, ^22^, ^23^, ^24^, ^25^–^30^ Of these, seven used Oswestry Disability Index.^17^, ^18^, ^22^, ^24^, ^28^–^30^ Three used Rolland Morris Disability Questionnaire 21, 23, 27 and one utilized Quebec Low Back Pain Disability Index.^26^ Compared to conventional exercise, core stability exercise resulted in significant improvement in functional status.

## Discussion

This review, which included fourteen studies, 17–30 compared the effects of core stability and conventional exercises on chronic low back pain. The risk of bias was examined for each article using the Cochrane collaboration recommendations. The results of this review indicate that core stability exercise is better than conventional exercise for pain relief and improving back specific function in the short term however Intermediate and long-term effects were not determined as there were no follow-up periods beyond three months. The primary results of this review are consistent with the findings of a meta- analysis of core stability exercise versus general exercise for chronic low back pain.^13^ The results of the meta-analysis indicated that core stability exercises are more effective than other types of exercise in improving back-specific functional status in the short term. Four other systematic reviews^31^–^34^ also reported that specific stabilization exercise was better than ordinary medical care and treatment by a general practitioner for reducing pain over the short term and intermediate term. Compared to the prior reviews, all the articles included in the current study were new, spanning from 2010 to 2020, and all of the articles in the current analysis considered only patients with chronic LBP (duration of pain >12 weeks). Based on these characteristics, this review is considered to be robust. Core stability is the ability to control the position and movement of the central portion of the body.^35^ Popular fitness programs, such as Tai Chi, Yoga, and Pilates, are based on core stability exercise principles. There are several different approaches currently in use for core stability exercise for LBP, which could lead to different results. A systematic review of different core stability exercises for LBP should be conducted to determine the optimal treatment approach.

## Limitation

The quality of this review may have been affected negatively due to inability to include other studies that were reported in other languages other than English and the year of publication which was restricted to the year 2010 and 2021. Numerous articles did not contain adequate information for evaluating the quality and clinical relevance of the data. Another limitation was the probability of publication bias, which we attempted to diminish via a substantial database search. However, unpublished articles were not searched.

## Conclusion

In this study, core stability exercise and conventional exercises for the lumbar region were both found to be beneficial in remission of pain and back specific function on chronic low back pain in the short term however; only two of these studies carried out follow up assessment for a period of three month which also showed that core stability was still more beneficial. Although this review may not be comprehensive however, it demonstrates deeper understanding of the use of core stability and conventional exercises in the management of CLBP.

## Figures and Tables

**Table 3:** Outline of each studies Physiotherapy evidence database score

Article	Random allocation	Allocation concealment	Blinding of participants /Therapist/ Accessor	Participants characteristics	Measure of outcome	Intention to treat analysis	Statistical comparison	Measurable variable
Javadian et al^17^ 2012	YES	UNCLEAR	NO/NO/NO	NO	YES	YES	YES	YES
You et al^18^ 2013	YES	YES	YES/YES/YES	YES	YES	YES	YES	YES
Ebrahimi et al^19^ 2014	YES	UNCLEAR	UNCLEAR/NO/NO	NO	YES	YES	YES	YES
Cho et al^20^ 2014.	YES	UNCLEAR	NO/NO/NO	YES	YES	YES	YES	YES
Alp et al^21^ 2014.	YES	YES	YES/NO/YES	YES	YES	YES	YES	YES
Cho et al^22^ 2015.	YES	UNCLEAR	NO/NO/NO	YES	YES	YES	YES	YES
Reddy et al^23^ 2015	YES	NO	NO/NO/NO	NO	YES	YES	YES	YES
Akhtar et al^24^ 2017.	YES	YES	YES/NO/YES	YES	YES	YES	YES	YES
Ali et al^25^ 2017.	YES	YES	UNCLR/NO/NO	YES	YES	YES	YES	YES
Ghorbanpour et al^26^ 2018	YES	YES	YES/UNCLR/UNCLR	YES	YES	YES	YES	YES
Noormohammadpour et al^27^ 2018	YES	YES	YES/NO/YES	YES	YES	YES	YES	YES
Waseem et al^28^ 2018.	YES	YES	YES/NO/NO	YES	YES	YES	YES	YES
Akter et al^29^ 2020	YES	YES	YES/YES/YES	YES	YES	YES	YES	YES
Yangma et al^30^ 2021.	YES	NO	UNCLR/NO/NO	YES	YES	YES	YES	YES

## Implications for Research

Articles that are methodologically sound and sufficiently powered are required to confirm the effects of core stability exercises and conventional exercises on pain relief and functional improvements in patients with chronic LBP. More so, the effects of core stability exercise and conventional exercises should be evaluated over a period of time post intervention.
